# Adolescents’ nutritional status and its association with academic performance in South Ethiopia; a facility-based cross-sectional study

**DOI:** 10.1186/s40795-021-00420-8

**Published:** 2021-04-29

**Authors:** Selamawit Woldeyohanes Katiso, Amene Abebe Kerbo, Samson Kastro Dake

**Affiliations:** Department of Reproductive Health and Nutrition, Wolaita Sodo University, College of Health Science and Medicine, School of Public Health, Wolaita Sodo, Ethiopia

**Keywords:** Academic performance, Adolescents, Nutritional status, Height for age, BMI for age, Wealth index

## Abstract

**Background:**

Adolescence is a particularly vulnerable stages of life in which malnutrition inhibits academic performance through poor growth and development, mental retardation, poor overall cognitive function and poor health status. However, there is a dearth of evidence regarding the association between nutritional status and academic performance among adolescent students. Therefore, this study aimed to determine the association of nutritional status and academic performance among adolescent secondary school students in Wolaita Sodo town, Southern Ethiopia.

**Methods:**

A facility-based cross-sectional study was conducted among 670 systematically selected adolescents in secondary schools of Wolaita Sodo town from April to June 2019. The academic performance of the adolescents was measured using the mean mark score of two consecutive semesters’ results of all subjects. Data were analyzed using Stata software Version 15. Descriptive statistics, binary and multiple linear regression analysis were done. Statistical association of dependent and independent variables was declared at *p-*valu*e* < 0.05.

**Results:**

The mean academic performance of students was 69.21 ± 0.42 (95% CI: 68.34–70.02%). A mean mark score of students increased by 1.89 (β = 1.89; 95%CI: 1.14, 2.64) for a unit increase in BMI for age z-score. Being female decreased a mean mark score by 2.63 (β = − 2.63; 95%CI: − 4.28, − 0.98) and being from a separated parents decreased by 4.73 (β = − 4. 73; 95%CI: − 6.73, − 2.74). A mean mark score of students from the first wealth class decreased by 9.92 (− 9.92; 95%CI: − 12.79, − 7.04) as compared to students from the highest wealth class. Attending private schools increased the mean mark score of students by 4.18 (β = 4.18; 95% CI: 2.46, 5.90).

**Conclusions:**

Interventions targeted at adolescents’ nutritional status should be designed and implemented. The town education office and concerned bodies **s**hould launch a school feeding program for public schools. Development and income generation activities should target households in the first wealth status. Schools are recommended to design additional teaching and learning schemes such as tutorial classes for girl students.

**Supplementary Information:**

The online version contains supplementary material available at 10.1186/s40795-021-00420-8.

## Background

Adolescent population comprises one-sixth of the world’s population; of which over 90% live in Sub-Saharan Africa and South and Southeast Asia [1]. Successful transition of adolescence to adulthood has a significant role in reducing problems lie ahead in the future and in breaking the intergenerational cycle of poverty [2].

Nutritional status is one of the main factors that could affect academic achievement by limiting students’ ability to learn [3]. Despite the economic growth observed in many developing countries, undernutrition continued to be highly prevalent underlying cause of poor health status and poor academic attainments’ [4].

Several studies were conducted on the magnitude and consequences of nutritional status of adolescent students [5–7]. According to the Global School-Based Student Health Survey, the mean Body Mass Index (BMI) estimates adolescents in South Asia, Southeast Asia, East Africa, West Africa and Central Africa is < 20. The lowest BMIs were seen in Ethiopia, Niger, Senegal, India, Bangladesh, Myanmar, and Cambodia [8]. On the other hand, globally, 10% of adolescents are overweight and obese with the prevalence of overweight and obesity varying between 2 and 3%. The prevalence ranges from 10% in Africa and Asia to more than 20% in the United States of America (USA) and Europe [9].

.According to the World Bank report, academic performance of students of Sub-Saharan African countries is less than half of what is expected for their age [10]. Ethiopia is among the countries where adolescent students’ academic achievement is unacceptably low [11]. Adolescence is one of the vulnerable stages of life where absolute nutrient needs are greater than that of infancy or childhood [12]. Undernutrition inhibits academic attainment through poor growth and mental development, reduced motivation and poor cognitive development [13, 14] while, ooverweight and obesity has the potential to impair academic performance via social pathways such as discrimination and stigma [15].

Research evidences also show that academic performance is affected by factors such as the wealth status of the parents, the type of school, parents’ educational status, marital and occupational status of the adolescents [16–19]. However, the association between nutritional status and the academic performance of the adolescent students in Ethiopia is left unknown fully. Therefore, the aim of this study was to assess the association of nutritional status with academic performance among adolescent students in Wolaita Sodo town, Southern Ethiopia.

## Methods

### Study setting, study design and study period

Wolaita Sodo town is the administrative capital of Wolaita Zone administration in Southern Ethiopia located at 380 km South from Addis Ababa. The town has 3 sub-cities and 11 lower administrative units. The total population of the town is estimated to be 182,607; from which 49% are females [20]. According to the information obtained from Wolaita Zone Agriculture and education departments, the common staple foods in the area are cereals, roots, tubers, and vegetables and there are two private and five public secondary schools in the town respectively. A facility-based cross-sectional study was conducted among adolescent students from April to June, 2019.

### Population and sampling

The source population for this study was all adolescents of secondary schools of Wolaita Sodo town and the study population are adolescent students of the selected schools. Pregnant adolescent girls, adolescents who were ill at the time of the study and with physical or visual disability were excluded from the study. A single population proportion formula was used to calculate the sample size with the following assumptions; 95% confidence level, 5% margin of error, an estimated magnitude of students’ academic performance of 72.8% taken from a similar study in Ethiopia [21], design effect of 2 and 10% non-response rate and the final sample size calculated is 670. There are seven schools in Wolaita Sodo town, two private and five public, and were stratified into public and private by assuming socio-economic differences among the families of the students and differences in teaching and learning resources between public and private schools. Among the seven secondary schools, one private and three public were randomly selected. The total sample size was allocated to the schools proportional to the number of students in each selected school. The study participants were selected by systematic sampling technique using the list of students enrolled in each school as a sampling frame. The sampling interval was determined by dividing the total number of students in the respective school grade level by the allocated sample size and was found to be five. The first participant was selected randomly by the lottery method, and then every fifth adolescent student was included in the study.

### Data collection

Data were collected using a structured interviewer-administered questionnaire. The questionnaire was developed and adopted from the Ethiopian Demographic and Health Survey (EDHS) validated tool and other related literature reviews [22–24] (Additional file 1). The questionnaire was pre-tested on 5% of the sample size on adolescent students from schools which were not selected for the actual data collection but no modification has been made. The data were collected by four data collectors and two supervisors after training was given for 2 days on the objective of the study, data collection procedures, anthropometric measurements, the confidentiality of the information and participant rights.

To ensure the reliability of anthropometric measurements, standardization test was done on five participants prior to actual data collection. First, the expert has taken the measurements and then the data collectors repeated the measurements on the same participants with some time intervals. The collected data were entered into ENA SMART software to check relative Technical Error of Measurements (TEM) and was found to be in the acceptable range, < 2.0%. Weight was measured using a portable digital flat Seca scale (Scale electronic scale, 770 Hamburg). Height was measured by Seca body meter (Seca 274 body meter). All measurements were taken three times, and the average was recorded as the final measurement. Academic performance and absenteeism data were taken from respective schools’ records.

### Variables

#### Dependent variable

The academic performance of the students which was calculated using two consecutive semesters mean mark scores out of 100.

#### Independent variables

##### Socio-economic and socio-demographic variables

Age of the adolescents, sex of adolescents, marital status of parents, educational and occupational status of parents and wealth status of the adolescents households. Wealth status was generated by using principal component analysis (PCA) and based on the results household wealth index/status was converted into quartiles and categorized as First, Second, Middle, Fourth, and Highest [22].

##### Nutritional status measurements and indices

**Underweight**- is BMI for age z-score (BAZ) of < − 2 standard deviation (SD) on the WHO growth reference cut-off point [25].

**Overweight**- was computed with BMI for age z-score (BAZ) of > + 1 SD on the WHO growth reference cut-off point [25].

**Obesity**- was computed with BMI for age z-score (BAZ) of > + 2 SD z-score based on the WHO reference cut-off point [25].

**Stunting**- is the height for age z-score (HAZ) of <− 2 SD on the WHO growth reference cut-off point [25].

##### Dietary diversity score

Dietary diversity was determined by using the Dietary Diversity Score (DDS). Three non-consecutive days 24-h recall of adolescents’ consumption of commonly consumed foods in the area was used to collect information on the DDS [23]. Foods were categorized into 10 groups based on FAO recommendations [1]; starch stable food [2], vegetables, 3) fruits [4], meat [5], egg [6], fish and other sea foods [7], legumes, nuts and seeds [8], milk and milk products [9], oil and fats [10], sweets, spices, condiments and beverage [26]. The response categories were “Yes” if at least one food item in the group was consumed and “No” when a food item in the group was not consumed. The results were summed and classified into < 4 food items and > 4 food items [27].

##### Behavioural factors

Alcohol consumption, the purpose of spending much time on the internet and being absent for 10% or more of school days for any reason in a calendar year.

### Data management and analysis

Data were entered into Epi-Data version 3.1 and analyzed using Stata version 15 statistical software. Anthropometric data were analyzed using the WHO Anthro-plus software version 1.0.4 and nutritional status of the adolescents was determined using WHO reference 2007 cut-off point [28]. Normality assumption was assessed for the dependent variable and the data were normally distributed (*p*-value is 0.77). Descriptive statistics such as frequencies, percentages, mean and standard deviation of the mean were done. Binary and multiple linear regression analysis were conducted to check the association between the dependent and independent variables. Variables with a *p*-value of less than 0.25 in the binary linear regression analysis were candidate variables for multiple linear regression analysis. Variables with the *p*-value < 0.05 in the multiple linear regression analysis were considered as statistically significantly associated with the dependent variable and parameter estimate (ß) with its 95% CI was reported.

## Results

### Socio-demographic characteristics

In this study, a total of 670 adolescents participated making the response rate of 100%. The mean age of the respondents was 16.2 ± 1.7. Of the total respondents, 50.6% were girls. The majority (81.3%) of the parents were currently married. More than one-fourth (27.3%) of the mothers and 47.5% fathers of the students completed college or university education. More than one-third (34.8%) of the mothers were merchants, while 42.4% of the fathers were government employees. Regarding the wealth index, 23.6%, of the study participants were from the fourth class households (Table 1).

**Table 1:** Socio-demographic and economic characteristics of the students and their parents involved in the study at Wolaita Sodo town in South Ethiopia, June 2019

Variables (*n* = 670)	Frequency	Percentage
Age in years		
10–14	137	20.5
15–19	533	79.6
Sex		
Male	331	49.4
Female	339	50.6
Current marital status of parents		
Married	545	81.3
Separated	125	18.7
Educational status of mothers		
No formal education	57	8.5
Read and write	134	20.0
Primary	123	18.4
Secondary	173	25.8
College & above	183	27.3
Educational status of fathers		
No formal education	21	3.1
Read and write	83	12.4
Primary	98	14.6
Secondary	150	22.4
College / university completed	318	47.5
Occupational status of the father		
Farmer	96	14.3
Merchant	158	23.6
Government employer	284	42.4
Private	115	17.2
Daily labourer	17	2.5
Occupational status of the mother		
Housewife	205	30.6
Merchant	233	34.8
Government employer	158	23.6
Private	66	9.9
Daily labourer	8	1.2
Wealth quintile		
First	110	16.4
Second	136	20.3
Middle	136	20.3
Fourth	158	23.6
Highest	130	19.4
School type		
Public	444	66.3
Private	226	33.7

### Nutritional status, dietary diversity and behavioural factors

The overall prevalence of any form of malnutrition was 29.3%; 6.3% (95% CI: 4.5, 8.5) were underweight, 9.7% (95% CI: 7.6, 12.2) overweight, 4.1% (95% CI: 2.8, 5.7) obese, and 9.2% (95% CI: 7.2, 11.4) were stunted. The majority (76.4%) of the adolescents spend much their time on the internet for social media purpose and about one-fourth (24.8%) reported drinking alcohol at least once before the study. About (24.3%) of the adolescents were absent for 10% or more of school days in the studied academic year. More than half (59.0%) of the adolescents had a dietary diversity score of ≤4 food items (Table 2).

**Table 2:** Nutritional status, dietary diversity, and behavioural characteristics of students involved in the study at Wolaita Sodo town in South Ethiopia, June 2019

Variables (*n* = 670)	Frequency	Percentage
Nutritional status (BAZ)		
Underweight	42	6.3
Normal	535	79.9
Overweight	65	9.7
Obese	28	4.2
Dietary diversity		
> 4	274	40.9
≤4	396	59.1
The purpose of spending time on the internet		
For academic purpose	144	23.5
For social media	469	76.5
Alcohol consumption		
Yes	166	24.8
No	504	75.2
Absent for 10% of school days in the studied academic year		
Yes	163	24.3
No	507	75.7

### Description of participants according to their academic performance

More than three-fourth (74.7%) of the adolescents aged 15–19 years performed below the mean academic score. Nearly one-fifth (79.2%) of the boys and 68.7% of the girls had below the mean academic performance respectively. More than half (56.4%) of the respondents who live in the first wealth quintile performed below the mean. The majority (89.2%) of those who live in a household with the highest wealth quintile performed above the mean academic score. The majority (88.1%) of the respondents who attended public schools and 66.7% who attended private schools academic performance was below the mean score. Fifty six (88.9%) of the overweight and 89.3% obese respondents performance was below the mean academic score. Two hundred twenty eight (83.2%) of the participants with a dietary diversity of more than four and 71.9% who spend their time on the internet for social media purposes performed below the mean academic score. Three-fourth (75.2%) of the participants who consume alcohol also performed below the mean (Table 3).

**Table 3:** Description of the study participants according to the academic performance at Wolaita Sodo town in South Ethiopia, June 2019

Variable (***n*** = 670)	Characteristics	Academic performance	
		Above the mean	Below the mean
Age in years	10–14	40 (29.2)	97 (70.8)
	15–19	135 (25.3)	398 (74.7)
Sex	Male	69 (20.9)	262 (79.2)
	Female	106 (31.3)	233 (68.7)
Wealth quintile	First	48 (43.6)	62 (56.4)
	Second	81 (59.6)	55 (40.4)
	Middle	114 (83.8)	22 (16.2)
	Fourth	136 (86.1)	22 (13.9)
	Highest	116 (89.2)	14 (10.8)
School type	Public	27 (11.9)	199 (88.1)
	Private	148 (33.3)	296 (66.7)
Nutritional status (BAZ)	Underweight	25 (58.1)	18 (41.9)
	Normal	140 (26.1)	396 (73.9)
	Overweight	7 (11.1)	56 (88.9)
	Obese	3 (10.7)	25 (89.3)
Dietary diversity	> 4	46 (16.8)	228 (83.2)
	≤4	129 (32.6)	267 (67.4)
The purpose of spending much time on the internet	For academic purpose	27 (18.8)	117 (81.3)
	For social media	132 (28.1)	337 (71.9)
Alcohol consumption	Yes	50 (30.1)	116 (69.9)
	No	125 (24.8)	379 (75.2)
Absent for 10% school days in the studied academic year	Yes	35 (21.5)	128 (78.5)
	No	140 (27.6)	367 (72.4)

### Proportion and predictors of academic performance

The mean academic performance of the students was 69.2 ± 11.0 SD (95% CI: 68.4, 70.0%) out of hundred. Being a girl decreased the mean score of academic performance by 2.6 (β = − 2.6; 95% CI: − 4.3, − 0.9). The mean score of students from separated parents decreased by 4.7 (β = − 4.7; 95% CI: − 6.7, − 2.7) as compared to students from married parents. Being from the first-class wealth index decreased the mean score of students by 9.9 (β = − 9.9; 95% CI: − 12.8, − 7.0). The mean mark score of students from a wealth index of the second class decreased by 5.7 (− 5.7; 95% CI: − 8.1, − 3.2) as compared to students from the highest wealth class. Attending private schools increased the average mark score of students by 4.2 (β = 4.2; 95% CI: 2.5, 5.9) compared to their counterparts. BAZ was positively associated with academic performance. A unit increase in BAZ increased the mean mark score of students by 1.9 (β = 1.9; 95% CI: 1.1, 2.6) (Table 4).

**Table 4:** Predictors of Academic Performance of adolescents in Secondary school at Wolaita Sodo town in South Ethiopia, June 2019

Variable (*n* = 670)	Binary linear regression		Multiple linear regression	
	Β	95% CI	Β	95% CI
Age				
15–19	2.5	0.4, 4.5	1.4	−0.4, 3.3
Sex				
Female	−2.0	−3.6, − 0.3	−2.6	−4.3, − 0.9
Marital status of parents				
Separated	−8.4	− 10.4, −6.3	− 4.7	−6.7, − 2.7
Education status of the mother				
Read and write	− 2.9	−5.0, − 0.9	− 1.7	− 4.9, 1.5
Primary	0.3	−1.8, 2.4	0.3	− 3.1, 3.7
Secondary	2.5	0.6, 4.4	−0.2	−3.6, 3.2
College & above	1.3	−0.6, 3.2	−3.5	−7.3, 0.3
Education status of the father				
No formal education	−1.4	−6.1, 3.4	2.5	−2.3, 7.3
Read and write	−3.6	−6.1, −1.1	1.5	−1.4, 4.3
Primary	−1.2	−3.6, 1.1	0.8	−1.7, 3.2
Secondary	0.2	−1.8, 2.2	−0.1	−2.1, 1.9
Occupational status of Mother				
Housewife	−1.2	−3.0, 0.6	0 .5	−2.1, 3.1
Merchant	−0.4	−2.1, 1.4	−0.01	−2.4, 2.4
Private employee	0.9	−1.9, 3.7	0.4	−2.4, 3.2
Daily labourer	−5.1	−12.7, 2.5	1.8	−5.6, 9.3
Wealth index				
First-class	−10.7	− 12.7, −8.6	−9.9	− 12.8, −7.0
Second class	−4.1	−6.2, −2.1	− 5.7	− 8.1, −3.2
Middle class	2.2	0.1, 4.3	−1.8	−4.1, 0.5
Fourth class	4.2	2.2, 6.2	−0.6	−2.9, 1.6
School type				
Private	5.83	4.1, 7.5	4.2	2.5, 5.9
The purpose of spending time on the internet				
For social media	−2.71	−4.8, −0.7	−1.4	−3.2, 0.4
Alcohol consumption				
No	1.79	−0.1, 3.7	−0.03	−1.8, 1.7
DDS				
Adequate	3.56	1.9, 5.2	1.01	−0.6, 2.6
Nutritional status				
BAZ	2.14	1.5, 2.8	1.89	1.1, 2.6
HAZ	1.33	0.6, 2.1	1.48	0.7, 2.2

## Discussion

This study has attempted to determine the association between nutritional status and academic performance among adolescent students. The result of the current study shows the mean academic score of students was 69.2 ± 1 (95% CI: 68.3, 70.0%). The study findings also revealed that being a girl student, nutritional status measures (BAZ and HAZ), being from the first-class wealth index household, attending private schools and separation of parents were statistically significantly associated with the academic performance of the students.

In this study, the mean academic score of the students was 69.2 ± 1 (95% CI: 68.3, 70.0%). This result is consistent with a study from Hawa Gelan (Ethiopia) where the mean academic performance was (67.2 ± 15.4%) [24]. However, the result is higher when compared with a study result of Nigeria which reported the mean academic performance of (53.3 ± 7.2) [29]. The result of this study is lower than the other study result in Debre-Tabor (Northern Ethiopia) where the mean academic score was (71.7 ± 12.6) [30]. This difference may be owing to the differences in the students’ assessment techniques, the curriculum and teaching-learning resources availability and accessibility. The other possible reason for the difference might be, in Ethiopia, although there exists nationally standardized testing system, the test is given only for 8th and 12th grades and the testing scheme and types of the tests depend on the school and the teachers.

There was a statistically significant and positive association between nutritional status (HAZ) and academic achievements. This finding agrees with studies from Northern and Southeast Ethiopia, where HAZ was associated with students’ academic performance [30, 31]. Despite the agreement with these studies, the correlation coefficient in the current study is relatively low. The possible reason might be the small sample size used in the mentioned studies. This finding is inconsistent with a study finding of Meskan District in Southern Ethiopia, where the study reported the absence of a statistically significant association between HAZ and academic performance [32].

In this study, the nutritional status measure (BAZ) is also statistically positively and significantly associated with the academic performance of the students. This result is not in line with another study conducted in North Ethiopia where the study reported that there was no statistical association between BAZ and academic performance [30]. This might be due to differences in sample size or variation of the variables considered during analysis.

In the current study, being a girl student decreased academic performance, this result is reported similarly in studies conducted in Ethiopia, Kenya, and Nigeria [33–35]. ,The most possible reason could be the differential and higher workload, lack of time to work on the assignments and shortage of time to eat meals timely and adequately among girls in the households when compared to boy students. A different finding was reported from Ghana where the academic achievement of girls was significantly higher than boys [36]. This difference might be due to the socio-cultural difference in the study settings.

The findings of the present study also disclosed that separation of parents has significantly lowered the academic performance of students when compared with students whose parents are in marital union. This result is consistent with the results of studies conducted in Addis Ababa (Ethiopia) and Ghana [37, 38]. This could be due to psycho-social and financial crises caused by separation or divorce the associated parental instability.

Academic performance of the students from a household of first wealth index or second wealth index class family was decreased students’ when compared to the students from the highest wealth index household families, This finding is in agreement with studies conducted in Dessie (Northwest Ethiopia) and Hawa Gelan district in Southwest Ethiopia [24, 39]. Similarly, another study from Goba town in Ethiopia depicted that a higher wealth index is associated with better mathematics scores [31]. This might be explained by the enabling environment created by providing educational materials and other resources which could have motivated students.

In the present study, a significant difference in academic performance among students attended private and a public school was found. Attending private schools increased the mean mark score of students as compared to their counterparts. This finding is in line with a study finding of Northwest Ethiopia [40]. Studies conducted in Nigeria and India also revealed that students who attended private schools scored better in reading, writing and mathematics as compared to students from public schools [41, 42]. This difference might be attributed to private schools better equipment in a library and laboratory facilities, regular and tight monitoring and evaluation of the teaching and learning process and students who attend private schools are mostly from a well to do families to provide better, adequate and timely nutrition than students of public schools.

### Limitations

The study sample consisted of adolescents students in Wolaita Sodo town secondary schools and therefore, the study results cannot be generalized to other schools elsewhere in Ethiopia or other Sub-Saharan Africa or other developing countries. The mean academic score used to assess the academic performance of the students is also not from standardized testing across the whole country. Thus, it might be difficult to extrapolate the proportion to the overall adolescent population in the country. We used cross-sectional data and the estimate might be better represented if longitudinal follow-up data were used. In this study, only anthropomorphic measurements were used to determine the nutritional status and did not assess the micronutrient status and its possible association with the academic performance of study participants. In the present study, other covariates such as cigarette smoking and time devoted to physical exercise have not been assessed. Furthermore, this study did not assess Intelligence Quotient (IQ) test due to a lack of standardized, culturally appropriate and contextualized testing systems in Ethiopia.

## Conclusions

This study ascertained poor academic performance was reported among female sex adolescent students, students whose parents were separated, and students of the first or second wealth index status households. Better academic performance was also seen in students with better nutritional status indicators such as BAZ and HAZ.

Wolaita Sodo town health office should design interventions targeted at improving adolescents’ nutritional status. A school feeding program should be launched particularly for underweight students. Microfinance institutions and other development and income-generation activities should target students from households of the first wealth status. Schools should give tutorial classes for girl students. Further studies to determine the association of nutritional status with school performance by including the micronutrient status data are recommended.

## Supplementary Information

**Additional file 1.** The Questionnaire.

## Data Availability

The datasets analyzed for this study are available with the corresponding author which can be accessed on reasonable request.

## Abbreviations

BAZ: BMI for Age Z-score
BMI: Body mass index
CI: Confidence interval
DDS: Dietary diversity score
FAO: Food and Agricultural Organization
HAZ: Height for Age Z-score
NGO: Non-Governmental Organization
NORHED: Norwegian Programme for Capacity Development in Higher Education and Research for Development
SD: Standard Deviation
SENUPH: South Ethiopia Network Universities in Public Health
TEM: Technical error of measurement
USA: United States of America
WHO: World Health Organization
WSU: Wolaita Sodo University
