# Supplementary material for: Adolescents’ nutritional status and its association with academic performance in South Ethiopia; a facility-based cross-sectional study
Source: BMC Nutr. 2021 Apr 29;7:15. doi: 10.1186/s40795-021-00420-8 (PMC8082903; doi:10.1186/s40795-021-00420-8)
Supplement: Supplementary file 1 — Additional file 1. The Questionnaire. [file 40795_2021_420_MOESM1_ESM.docx]

**Additional file 1****: The Questionnaire**

Section I: Questionnaire identification

1. Date (dd/mm/yy) ____/ ______/ _______
2. Kebele _________________________
3. Name of the school ___________________
4. Grade______ Section____________
5. Student’s code / Identification number __________
6. Interviewer Name______________________________ Sign_________date________
7. Supervisor Name______________________________ Sign_________date________

**Part I: Socio-Demographic and Socio Economic Questions**

| **S.N** |  | **Socio-Demographic information** |  | | | **Response** | **Skip** | | |  |
| --- | --- | --- | --- | --- | --- | --- | --- | --- | --- | --- |
| 101 |  | Age in year | __________________ | | | |  | | |  |
| 102 |  | Sex |  | 1. Male 2. Female | | |  | | |  |
| 103 |  | What is the current marital status of your parents |  | 1. Single 2. Married 3. Divorced 4. Widowed | | |  | | |  |
| 104 |  | What is the highest level of education your mother completed? |  | 1. No formal education  2. Can read and write  3. primary school  4. secondary school  5. College/University completed | | |  | | |  |
| 105 |  | What is the highest level of education your mother completed? |  | 1. No formal education  2. can read and write  3. primary school  4. secondary school  5. College/University completed | | |  | | |  |
| 106 |  | What is your father’s occupation? |  | | 1.Farmer  2.Merchant  3.Government employer  4.Private  5.Daily labourer | | |  |  | |
| 107 |  | What is your mother’s occupation? |  | | 1.House wife  2.Merchant  3.Government employer  4.Private  5.Daily labourer | | |  |  | |

**Part II: Wealth Index of Household’s Related Questions**

| **S.N** | **HHs information** | **Response** | **Skip** |
| --- | --- | --- | --- |
| 201 | What is your common source of drinking water | 1. Piped into dwelling 2. Public tap/ stand pipe 3. Protected well 4. Rain water 5. Bottled water 6. Surface water (river/lake/pond/stream/dam) 7. Other (specify) ___________ |  |
| 202 | In what kind of house do you live? | 1. Rent 2. Own |  |
| 203 | The common light of your house? | 1. Fire 2. Lantern 3. Beer cane with gas/candle 4. Torch light 5. Generator 6. Biogas 7. Solar lamp 8. Electricity 9. Others (specify)__________ |  |
| 204 | What are the building materials for the house floor? | 1. Earth/sand  2. Dung  3. Plastic  4. Wood/planks  5. Bamboo  6. Ceramic tiles  7. Cement  8. Other (specify)___________ |  |
| 205 | What are the building materials used for the wall of the house? | 1. Earth  2. Plastic  3. Wood  4. Local brick  5. Cement/Cement brick  6. Other (specify) ____________ |  |
| 206 | What are the building materials used for the inside ceiling of the house? | 1. No ceiling  2. Plastic  3. Cement/Cement brick  4. Cloth  5. Corrugated iron sheet  6. Wood  7. Other (specify) ___________ |  |
| 207 | What are the building materials used for the roof of the house? | 1. Thatch  2. Plastic  3. Wood  4. Corrugated iron sheet  5. Cement/ cement brick  6. Local brick  7. Other (specify) ____________ |  |
| 209 | What is the common material used for cooking? | 1. Electricity  2. Straw/shrubs/grass.  3. Charcoal  4. Liquid petroleum gas/natural gas/biogas  5. Animal dung  6. Other (specify) ___________ |  |
| 210 | Where do you cook food? | 1. No food cooked in the house  2. In a separate building/kitchen  3. In the living house 4. Others (specify)­ ___________ |  |
| 211 | What kind of toilet facility do you use? | 1. Open field  2. Pit latrine without slab/open pit  3. Pit latrine with slab  4. Cemented latrine  5. Flush/ pour flush to septic tank  6. Other/ specify ____________ |  |
| 212 | Do you share this toilet facility with other households? | 1. Yes  2. No |  |
| 213 | Is there vehicle to transport family from place to place? | 1. Yes  2. No |  |
| 214 | Do you have refrigerator in your home? | 1. Yes  2. No |  |
| 215 | Do you have television in your home? | 1. Yes  2. No |  |
| 216 | Do you have an electric mitad in your home? | 1. Yes  2. No |  |
| 217 | Is there bed in your parents’ home? If yes, what type? | 1. A bed with cotton 2. A bed with sponge 3. A bed with spring matters 4. Other /specific____________ |  |
| 218 | Do your parents own any agricultural land? | 1. Yes  2. No | If “No” skip to Q 227 |
| 219 | What type of agricultural land do your parents own? | 1. Private 2. Rent |  |
| 220 | Is there annual farm product per quintal? | 1. Yes 2. No |  |
| 221 | Wheat | 1. Yes 2. No |  |
| 222 | Barley | 1. Yes 2. No |  |
| 223 | Maize | 1. Yes 2. No |  |
| 224 | Shin bra | 1. Yes 2. No |  |
| 225 | Teff | 1. Yes 2. No |  |
| 226 | Cassava | 1. Yes 2. No |  |
| 227 | Do you own cattle? | 1. Yes 2. No | If “No” skip to part-III |
| 228 | Milk cows, oxen or bulls | ------------- in number |  |
| 229 | Horses, donkeys, or mules | ------------- in number |  |
| 230 | Goats /Sheep | ------------- in number |  |
| 231 | Chickens | ------------- in number |  |

**Part III: Questions Related with Behavioural Factors**

| **S.N** | **Questions** | **Response** | **Skip** |  |
| --- | --- | --- | --- | --- |
| 301 | Do you use/ consume alcohol? | 1. Yes 2. No | If you say “No” skip to Q 302 | |
| 302 | Do you use an internet? | 1. Yes 2. No | If you say “No” skip to Q part IV | |
| 303 | If you say ‘yes’ in Q 301, for what purpose do you spend more time? | 1. For academic purpose 2. For social media 3. Others (specify) ____________ |  | |
|  |  |  |  |  |
| 304 | Type of school | 1. Public  2. Private |  | |
| 305 | Absent for 10% school days in the studied academic year | 1. Yes  2. No |  | |

**Part IV: Dietary Diversity Questions**

Please describe the foods (meals and snacks) that you eat and drink yesterday during the day and night, you are kindly requested to start with the first food or drink of the morning.

Day 1: Day of week (circle the day): 01-Mon 02-Tue 03-Wed 04-Thu 05-Fri 06-Sat 07-Sun

| Time |  | Place Of Eaten | Food Description |  | Description Of Ingredients | |
| --- | --- | --- | --- | --- | --- | --- |
|  |  |  |  |  |  | |
| Breakfast |  |  |  |  |  | |
|  |  |  |  |  |  | |
| Snack (mid- morning) |  |  |  |  |  | |
|  |  |  |  |  |  | |
| Lunch |  |  |  |  |  | |
|  |  |  |  |  |  | |
| Snack (mid-afternoon) |  |  |  |  |  | |
|  |  |  |  |  |  | |
| Dinner |  |  |  |  |  | |
|  |  | |  |  | |  |

Day 2: Day of week (circle the day): 01-Mon 02-Tue 03-Wed 04-Thu 05-Fri 06-Sat 07-Sun

| Time |  | Place Of Eaten |  | | Food Description | |  | Description Of Ingredients | |
| --- | --- | --- | --- | --- | --- | --- | --- | --- | --- |
|  |  |  |  | |  | |  |  | |
| Breakfast |  |  |  | |  | |  |  | |
| Snack (mid- morning) |  |  |  | |  | |  |  | |
| Lunch |  |  |  | |  | |  |  | |
| Snack (mid-afternoon) |  |  |  | |  | |  |  | |
| Dinner |  |  |  | |  | |  |  | |
|  |  | | |  | |  | | |  |

Day 3: Day of week (circle the day): 01-Mon 02-Tue 03-Wed 04-Thu 05-Fri 06-Sat 07-Sun

| Time | |  | Place Of Eaten | | | Food Description | | Description Of Ingredients | | |
| --- | --- | --- | --- | --- | --- | --- | --- | --- | --- | --- |
|  | |  |  | | |  | |  | | |
| Breakfast | |  |  | | |  | |  | | |
|  | |  |  | | |  | |  | | |
| Snack (mid- morning) | |  |  | | |  | |  | | |
|  | |  |  | | |  | |  | | |
| Lunch | |  |  | | |  | |  | | |
|  | |  |  | | |  | |  | | |
| Snack (mid-afternoon) | |  |  | | |  | |  | | |
|  | |  |  | | |  | |  | | |
| Dinner | |  |  | | |  | |  | | |
|  | |  | | |  | |  | | |  |
| **Question number** | **Food group** | | | **Locally available food** | | | | | **Yes=1**  **No=0** |  |
| 401 | Cereals | | | Teff, maize ,wheat ,sorghum, rice, barley, emmer wheat  (eg, enjera, porridge , paste ,bread ,keta) | | | | |  |  |
| 402 | Vegetables | | | Cabbage, Ethiopian kale, carrot , Swiss chard, tomato, potato, pepper, onion, cucumber | | | | |  |  |
| 403 | Fruits | | | Mango, banana, papaya, avocado, Lemon, grape, pineapple, orange ,guava zeytune),peach(kok)and tangerine(mandarine) | | | | |  |  |
| 404 | Meat and meat products | | | Beef meat ,chicken ,goat meat, mutton  Roasted  (eg, ye beratibs, dorowat, ye fiyyaltibs,qiqqil and ye beg sigaqiqqil | | | | |  |  |
| 405 | Egg | | | eggs from chicken | | | | |  |  |
| 406 | Fish and other seafood | | | Fresh fish | | | | |  |  |
| 407 | Legumes, nuts, seeds | | | beans, peas, lentils, nuts, kidney beans (red Wolita)and chick peas (shimbera) | | | | |  |  |
| 408 | Milk and milk products | | | milk, cheese, yogurt, whey | | | | |  |  |
| 409 | Oil and fats | | | oil, fats or butter added to food or used for cooking | | | | |  |  |
| 410 | Sweets | | | sugar, honey, sweetened juice drinks, sugary foods such as chocolates, candies, cookies and cakes | | | | |  |  |
| 411 | Spices and beverage | | | Spices (cumin, black (tiqurazmud) pepper, cardamom(kororyima) salt),  Beverage ,coffee, tea, emmer wheat(ye ajjaatimyit) | | | | |  |  |
| 412 | Tubers and roots | | | sweet potatoes, taro, cassava, false banana(Enset)  (eg ,bulla ganfo,mucho,qocho) | | | | |  |  |

**Part V. Anthropometric Measurements**

| **No** | **Weight measurements(kg)** | | | | **Height measurements(cm)** | | | |
| --- | --- | --- | --- | --- | --- | --- | --- | --- |
| 501 | 1^st^ | 2^nd^ | 3^rd^ | The average | 1^st^ | 2^nd^ | 3^rd^ | The average |
|  |  |  |  |  |  |  |  |  |

**Part VI Academic Performance Data and absenteeism**

| **S.N** | **Academic performance** | | |
| --- | --- | --- | --- |
|  | Previous semester average score of all subjects | Current semester average score of all subjects | Both semesters average score of all subjects |
|  |  |  |  |
|  |  |  |  |
